# Conjugation of vascular endothelial growth factor to poly lactic-co-glycolic acid nanospheres enhances differentiation of embryonic stem cells to lymphatic endothelial cells

**DOI:** 10.5713/ajas.20.0202

**Published:** 2020-08-21

**Authors:** Hyunjin Yoo, Dongyoon Choi, Youngsok Choi

**Affiliations:** 1 Department of Stem Cell and Regenerative Biotechnology, Institute of Advanced and Regenerative Science, Konkuk University, Seoul 05029, Korea

**Keywords:** Stem Cell, Lymphatic Endothelial Cell, Poly Lactic-co-glycolic Acid (PLGA)

## Abstract

**Objective:**

Pluripotent stem cell-derived lymphatic endothelial cells (LECs) show great promise in their therapeutic application in the field of regenerative medicine related to lymphatic vessels. We tested the approach of forced differentiation of mouse embryonal stem cells into LECs using biodegradable poly lactic-co-glycolic acid (PLGA) nanospheres in conjugation with growth factors (vascular endothelial growth factors [VEGF-A and VEGF-C]).

**Methods:**

We evaluated the practical use of heparin-conjugated PLGA nanoparticles (molecular weight ~15,000) in conjugation with VEGF-A/C, embryoid body (EB) formation, and LEC differentiation using immunofluorescence staining followed by quantification and quantitative real-time polymerase chain reaction analysis.

**Results:**

We showed that formation and differentiation of EB with VEGF-A/C-conjugated PLGA nanospheres, compared to direct supplementation of VEGF-A/C to the EB differentiation media, greatly improved yield of LYVE1(+) LECs. Our analyses revealed that the enhanced potential of LEC differentiation using VEGF-A/C-conjugated PLGA nanospheres was mediated by elevation of expression of the genes that are important for lymphatic vessel formation.

**Conclusion:**

Together, we not only established an improved protocol for LEC differentiation using PLGA nanospheres but also provided a platform technology for the mechanistic study of LEC development in mammals.

## INTRODUCTION

The lymphatic system plays essential roles in the host defense mechanism including the promotion of immune cell maturation, lipid reabsorption, and balance of interstitial fluid [1]. Although lymphatic capillaries are located close to blood capillaries, the two systems are anatomically separated because the tips of lymphatic capillaries are blunted [2]. In previous studies employing mouse models, transcription factors such as SRY-box transcription factor 18 (Sox18) and CoupTFII modulated lymphatic endothelial cell (LEC) development by inducing the salt-and-pepper pattern of Prox1 (prospero homeobox 1) expression in a subset of endothelial cells (ECs) of the cardinal vein at E 9.0 to 9.5. Subsequently, the lineage-committed LEC progenitors migrated towards vascular endothelial growth factor (VEGF)-C, a high level of which was released mainly from the surrounding mesenchyme to form primitive lymphatic sacs [3–5]. The VEGF-C/vascular endothelial growth factor receptor 3 (VEGFR3) axis appeared essential for the processes of LEC specification [6] and migration as knockout of either VEGF-C or VEGFR3 in mouse models displayed embryonic lethality with aberrant lymphatic formation [6,7]. Consistent with this notion, the effects of VEGF-C/VEGFR3 led to the proliferation and migration of LECs and blood ECs (BECs) [8]. Furthermore, VEGF-A was found to promote proliferation of lymphatic vessels [9].

Previous studies demonstrated that LECs were successfully differentiated from both mouse and human pluripotent stem cells (PSCs) using VEGF-C/VEGFR3 signals [10,11]. A combined administration of VEGF-A and VEGF-C to embryoid bodies (EBs) maintained in either simple differentiation media or 3D collagen matrix enhanced LEC differentiation [11]. More recently, Kono et al [12] showed that the efficiency of human PSC-derived LEC differentiation could be maximized with the aid of a co-culture system in the presence of OP9 feeder, VEGF-A, VEGF-C, and epidermal growth factor (EGF). However, it was clear that PSC-derived LEC differentiation needed to be improved for therapeutic uses [13]. Moreover, because of scarcity of *in vivo* LEC samples, an optimized system for LEC differentiation was also needed to study the underlying mechanism of *in vivo* LEC development.

Biodegradable substrates containing poly lactic-co-glycolic acid (PLGA) are widely being used in tissue regeneration and stem cell-derived differentiation [14]. For example, heparin-conjugated PLGA nanospheres were shown to accelerate *in vivo* wound closure by enhancing angiogenesis [15,16]. One of the advantages of using PLGA nanospheres as the substrate is that it can release conjugated growth factors (GFs) in a sustainable manner [16,17]. Indeed, a sustained release of fibronectin after conjugation with 3,4-dihydroxy-L-phenylalanine (L-DOPA)-coated PLGA improved *in vitro* neuronal differentiation of human mesenchymal stem cells. This study highlighted the “ease-of-fabrication” of PLGA as a nanopatterned-PLGA with L-DOPA, significantly enhancing guided alignment of neuronal differentiation [18].

In this study, we analyzed the practical application of PLGA nanospheres in differentiation of LECs from mouse embryonal stem cells (mESCs) and found that mouse EBs conjugated with VEGF-A/C loaded heparin-conjugated-PLGA nanospheres enhanced LEC differentiation by enhancing the expression of the genes that are important for lymphangiogenesis. Therefore, our study not only established an improved protocol for LEC differentiation using PLGA nanospheres but also provided a platform technology for the mechanistic study of LEC development in mammals.

## MATERIALS AND METHODS

### Preparation of VEGF-A/C loaded heparin-conjugated PLGA nanospheres

Preparation of heparin-conjugated PLGA nanospheres has been described in previous studies [15]. The synthesized PLGA (molecular weight ~15,000) was used for heparin conjugation. Heparin-PLGA nanospheres were prepared using oil/water emulsion, according to the solvent evaporation extraction method. The heparin-conjugated PLGA nanospheres were morphologically examined under a scanning electron microscope (SEM, S-4300, Hitachi, Tokyo, Japan). Size distribution was evaluated using a dynamic laser light scattering instrument (Zetasizer 3000HS, Malvern, UK). Then, VEGF-A/C was loaded onto the size-homogeneous heparin-PLGA nanospheres (hereafter referred to as NP_AC_ [VEGF-A/C-conjugated nanoparticle]).

### Embryoid body formation and differentiation

mESCs (E14) were cultured in Dulbecco’s modified eagle medium (SH3002101; Hyclone, Chicago, IL, USA), supplemented with 15% fetal bovine serum (35-015-CV; Corning, New York, NY, USA), 1X penicillin-streptomycin (15140122; Gibco, Waltham, MA, USA), 1X GlutaMAX (35050061; Gibco, USA), 1 mM sodium pyruvate (11360070; Gibco, USA), 1X non-essential amino acid (11140050; Gibco, USA), 1X β-mercaptoethanol (21985023; Gibco, USA), and 10^6^ U of leukemia inhibitory factor (ESG1107; Merck, Darmstadt, Germany), on gelatin-coated dishes. The initial EBs were formed in hanging drops (500 cells/drop) in either differentiation media containing GFs (30 ng/mL VEGF-A [293-VE-010; R&D systems, Minneapolis, MN, USA] and 50 ng/mL VEGF-C [2179-VC-025; R&D systems, USA]) (hereafter referred to as EBGF) or differentiation media containing 60 μL/mL of NP_AC_ (hereafter referred to as EBNP_AC_). The released GF concentration from the NPAC was adjusted to reach ~30 ng/mL (VEGF-A) and ~50 ng/mL (VEGF-C) at differentiation day2 based on the study by La and Yang [15]. GF-free EB (hereafter referred to as EB) was also produced and used as the control group. Morphological analysis of EB was performed under an SEM. After 48 h of EB formation in hanging drops, 10 EBs/well were transferred to BioCoat Collagen I glass multi-well culture slide (354630; Corning, USA) and incubated for 8 d.

### Immunofluorescence

For immunofluorescence staining, attached cells on the slide were fixed with 4% paraformaldehyde solution at room temperature for 5 min, washed with 1X phosphate buffer saline (PBS), and permeabilized with 0.1% TritonX-100/PBS for 5 min. Cells were incubated in blocking solution (0.1% Tween20/PBS, 3% normal serum, and 2% bovine serum albumin) for 1 h and incubated again in primary antibodies (CD31 [550274; BD PharMingen, Franklin Lakes, NJ, USA] and LYVE-1 [11-034; AngioBio, San Diego, CA, USA]) at 4°C overnight. Slides were washed, incubated in secondary antibodies at room temperature for 1 h, washed again, fixed, and then mounted using mounting solution that contained 4′,6-diamidino-2-phenylindole. Z-stack images were captured using a confocal microscope (Zeiss, Oberkochen, Germany). Quantification of vessel length of CD31- or LYVE1(+) vessels and dots was performed using the ImageJ package. Total CD31 or LYVE1(+) pixel values were obtained from three biological replicas.

### Quantitative real-time polymerase chain reaction

Total RNA was isolated using the Ribospin Kit (304-150; GeneAll, Seoul, Korea), and cDNA was synthesized using the HyperScript First strand synthesis Kit (601-005; Geneall, Korea). cDNA was mixed with Fast SYBR Green Master Mix (4385612; Applied Biosystems, Foster City, CA, USA), and quantitative real-time polymerase chain reaction (qPCR) was performed using the StepOnePlus Real-Time PCR System (Applied Biosystems, USA). Glyceraldehyde-3-phosphate dehydrogenase was used for normalization. The primer sequences for qPCR were shown in Table 1.

### Statistical analysis

Lengths of CD31- or LYVE1(+) vessels and LYVE1(+) spots were quantified using the ImageJ software (1.8.0_112; NIH, Bethesda, MD, USA). All statistical analyses were performed using SigmaPlot (12.0; Systat Software, San Jose, CA, USA). Normality and equal variance tests were applied to all samples. One-way analysis of variance test was used for statistical analyses. Statistical significance was determined at p≤0.05.

## RESULTS

### NP_AC_-mediated embryoid body formation enhanced LEC differentiation

**Figure 1:**
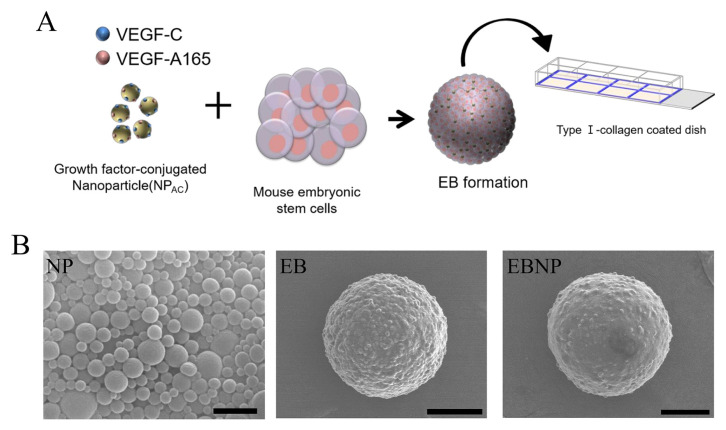
Characterization of EBNP_AC_. (A) Schematic diagram of the experimental procedure of lymphatic endothelial cells differentiation using EBNP_AC_. (B) Scanning electron microscopy image of embryoid bodies constituted with EBNP. Scale bar, 100 μm. EBNP_AC_, differentiation media containing 60 μL/mL of size-homogeneous heparin-PLGA nanospheres. EB, embryoid body; NP, nanoparticle; PLGA, poly lactic-co-glycolic acid.

**Figure 2:**
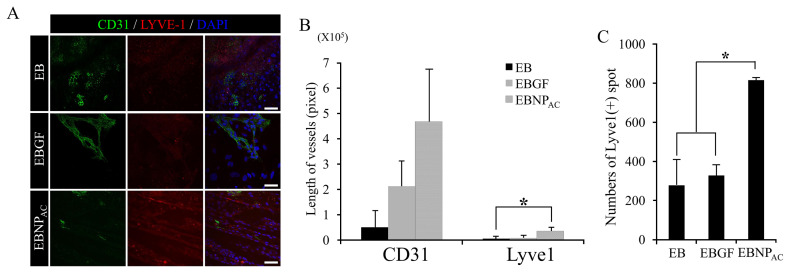
Analysis of the differentiation potential of EBNP_AC_ to lymphatic endothelial cells (LECs). (A) Immunofluorescent staining with blood endothelial cell (BEC) marker (CD31, green) and LEC marker (LYVE-1, red) after 8 d of differentiation. DAPI was used for nuclear counter staining. Scale bar, 50 μm. (B) Quantification of CD31- or LYVE1(+) vessels. Values represent mean±standard error mean (SEM). Asterisk represents p value <0.01. (C) Quantification of LYVE1(+) dots. Values represent mean±SEM. EBNP_AC_, differentiation media containing 60 μL/mL of size-homogeneous heparin-PLGA nanospheres. EB, embryoid body; NP, nanoparticle; DAPI, 4′,6-diamidino-2-phenylindole; PLGA, poly lactic-co-glycolic acid.

The amount of heparin conjugated to the nanosphere surfaces was determined using toluidine blue, and was 16 mg/g of PLGA nanospheres. This amount of heparin was sufficient to bind several GFs to the heparin binding site of heparin-conjugated PLGA nanospheres (data not shown). To assess the EB-forming ability of NP_AC_, 500 ESCs were mixed with NP_AC_ for 2 d (Figure 1A) and imaged with the SEM. As shown in Figure 1B, homogeneous EBNP (PLGA nanoparticle-only) was successfully formed, and no difference in sizes of EB and EBNP was observed. The same amounts of EBNP_AC_, EBGF, and EB were transferred into collagen-coated dishes and cultured for 8 d. Immunocytochemistry with CD31 and LYVE1 showed that the lengths of LYVE1(+) LECs in EBNPAC were significantly increased compared to those in EBGF and EB (Figure 2A, B). Furthermore, quantification of patched LYVE1(+) LECs in EBNP_AC_ was also significantly greater than that in EBGF and EB. CD31(+) BEC differentiation was markedly enhanced in EBNP_AC_ (Figure 2B, C). The data clearly demonstrated that the differentiation potential of NP_AC_ was superior to that of direct addition of GFs into differentiation media for LEC differentiation.

### LEC differentiation from mESCs was enhanced via overexpression of LEC-related genes after NP_AC_ treatment

**Figure 3:**
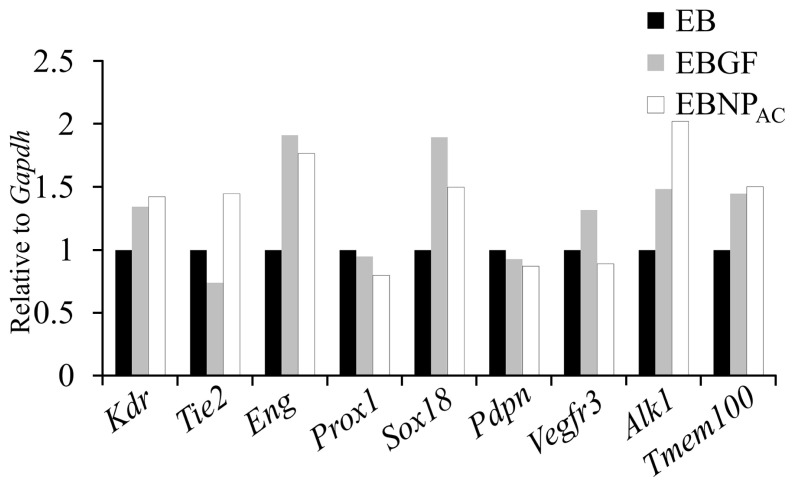
Quantitative real-time polymerase chain reaction analysis of expression of genes related to blood endothelial cell (*Kdr*, *Tie2*, *Eng*, and *Alk1*) or lymphatic endothelial cell (*Prox1*, *Sox18*, *Pdpn*, *VEGFR3*, and *Tmem100*) development. Expression of the genes in embryoid body was set as 1. *Gapdh* was used as the reference gene. *Kdr*, kinase insert domain receptor; *Tie2*, tyrosine kinase with immunoglobulin like and EGF like domains 2; *Eng*, endoglin; *Alk1*, activin receptor-like kinase 1; *Prox1*, prospero homeobox 1; *Sox18*, SRY-box transcription factor 18; *Pdpn*, podoplanin; *VEGFR3*, vascular endothelial growth factor receptor 3; *Tmem100*, transmembrane protein 100; *Gapdh*, glyceraldehyde-3-phosphate dehydrogenase.

We then examined the expression pattern of genes that are important for LEC differentiation. The qPCR analysis was performed using genes associated with vascular development and maintenance. As shown in Figure 3, although the level of *Prox1* did not change, levels of *Sox18* [19], Activin receptor-like kinase 1 [3,20], and transmembrane protein 100 (*Tmem100*) [21], the genes critical for lymphangiogenesis, and LEC development and identification, were elevated in EBNP_AC_. The data indicated that enhanced LEC differentiation using EBNP_AC_ was mediated by elevated expression of genes that are important for LEC development and function.

## DISCUSSION

In the present study, we determined a potential use of biodegradable PLGA nanospheres in conjugation with GFs (VEGF-A and VEGF-C) and forced differentiation of mESCs into LECs. Previously, PLGA sustainably released GFs over time, and could successfully be used in neuron differentiation of human neural stem cells [18]. Our study showed that nano-scale PLGA nanospheres did not affect EB formation and its attachment to type I collagen matrix during differentiation. Furthermore, our study showed that EBNP_AC_ enhanced LEC differentiation. Differentiation of mouse or human ESCs into cells of lymphatic lineage has been reported in several studies [10–12,22]. A previous study showed that LEC differentiation from mESCs was achieved after 18 d of differentiation in culture media in the presence of VEGF-A and VEGF-C. However, our study revealed that the use of EBNP_AC_ significantly shortened the LEC differentiation time by enhancing the expression of genes that are important for LEC development and differentiation.

Sox18/CoupTFII and VEGF-C/VEGFR3 axes were required for specification of LEC progenitors. For this, these axes activated Prox1 in cardinal vein ECs and lymphangiogenesis by enhancing migration of LECs and lymph sac formation, respectively [7,9,23,24]. In addition, function of other lymphatic factors, such as Sox18, Podoplanin, LYVE1, forkhead box protein C2 (Foxc2), and Tmem100, in regulating lymphatic development have been documented using mouse and zebra fish systems [19,21,23–25]. Impaired function of these genes was shown to be linked to certain types of lymphatic disorders. For example, *Sox18* and *Foxc2* mutations in humans cause hypotrichosis-lymphoedema-telangiectasia and lymphedema-distichiasis, respectively [26]. Mutation in the *VEGFR3* gene causes hereditary lymphedema type I (Milroy disease) [8]. Approximately 20% of cancer patients present with unwanted complications related to lymphatic edema [27]. However, the current strategy for treatment of lymphedema is diuretic therapy, reduction of swelling, and prevention of scarring and other complications. Therefore, cell-based therapy is now considered an unprecedented or alternative approach for treatment of this disorder. Our study demonstrated that *in vitro* LEC production by durable release of GFs from NP_AC_ was a simple and efficient method. This method could potentially be used for wound repair and lymphatic regeneration by accelerating *in vivo* lymphangiogenesis. Thus, using EBNP_AC_ in further *in vivo* studies is necessary to develop better and elaborated cell therapies against lymphatic disorders.

## Figures and Tables

**Table 1:** Primer sequences used for gene cloning and point mutation

Gene ID	Forward	Reverse
*Gapdh*	5′-CATGGCCTTCCGTGTTCCTA	5′-GCCTGCTTCACCACCTTCTT
*Kdr*	5′-AGCTCAGGCTTTGTTGAGGA	5′-AGATGCTCCAAGGTCAGGAA
*Tie2*	5′-AGACCCTTACGGGTGTTCCT	5′-CAGTGGCACCTGAGCTTACA
*Eng*	5′-TTCGTACAGGTGAGCGTGTC	5′-GGATGAGTTCCACCATGTCC
*Prox1*	5′-GAGCTATACCGAGCCCTCAA	5′-ACTCCCGTAACGTGATCTGC
*Sox18*	5′-CTTCATGGTGTGGGCGAAG	5′-TCAGCTCCTTCCACGCTTT
*Pdpn*	5′-CCATGATCACAGAGAACACGA	5′-CGTTTCATCCCCTGCATTAT
*VEGFR3*	5′-TAGGAGGAGACCTGGAAGCA	5′-GGTCCTCAGCTTCTTGGACA
*Tmem100*	5′-GTGCCGAACTCTCCTGCTAC	5′-ATGGAACCATGGGAATTGAA

*Gapdh*, glyceraldehyde-3-phosphate dehydrogenase; *Kdr*, kinase insert domain receptor; *Tie2*, tyrosine kinase with immunoglobulin like and EGF like domains 2; *Eng*, endoglin; *Prox1*, prospero homeobox 1; *Sox18*, SRY-box transcription factor 18; *Pdpn*, podoplanin; *VEGFR3*, vascular endothelial growth factor receptor 3; *Tmem100*, transmembrane protein 100.
